# Adult-Onset Still’s Disease Associated With Hyperimmunoglobulin-E: A Case Report

**DOI:** 10.7759/cureus.86063

**Published:** 2025-06-15

**Authors:** Parishad Yakhchalian, Mahwish Sheikh, Mohammed G Elhassan

**Affiliations:** 1 Internal Medicine, Saint Agnes Medical Center, Fresno, USA

**Keywords:** adult-onset still's disease, aosd, autoimmune disease, hyperimmunoglobulin, quotidian fever

## Abstract

Adult-onset Still's disease (AOSD) is a rare, chronic, multisystem inflammatory disorder of unknown etiology. Diagnosis is made clinically and can be challenging. AOSD typically affects young adults but can occur in older patients. The association between AOSD and very high immunoglobulin-E levels (also known as hyperimmunoglobulin-E) is not well studied. In this report, we present the case of a middle-aged woman with AOSD who presented with more than two weeks of fever, severe generalized weakness, and sore throat, and responded very well to prednisone. She was found to have hyperimmunoglobulin-E without a history of allergic disease or parasitic infection.

## Introduction

Adult-onset Still's disease (AOSD) is a chronic multisystem inflammatory disorder of unknown etiology with an estimated prevalence of 1-10 cases per million [1,2]. Although typically affecting individuals between 16 and 35 years of age, AOSD has also been reported in older adults. Per Yamaguchi's criteria, which is the most common criterion used for diagnosis, the clinical presentation of AOSD includes four main features: high spiking fever (> 39°C), arthralgia/arthritis, evanescent skin rash, and leukocytosis ≥ 10,000 cells/μL with neutrophils ≥80% [3]. Other “minor” features include: sore throat, lymphadenopathy, hepatomegaly or splenomegaly, abnormal liver function tests, and absence of rheumatoid factor and antinuclear antibody. Marked hyperferritinemia, particularly low glycosylated ferritin, may support the diagnosis, though it is not pathognomonic. Additional symptoms may occur, making the diagnosis more challenging, particularly in older individuals. Older patients may exhibit distinct clinical features, including higher complication rates and reduced survival [4,5]. However, the number of older age-onset AOSD cases remains insufficient to fully explore their clinical features. 

The aims of reporting this case are to describe a rare presentation of AOSD in a middle-age woman with quotidian fever, severe sore throat, and transient skin rashes which initially mimicked an infectious process for over two weeks before admission, and was associated with very high level of immunoglobulin (Ig)-E (also known as hyperimmunoglobulin-E), which seems to be an unusual feature of the disease.

## Case presentation

A 55-year-old female patient with a history of hypertension and diabetes mellitus presented to the emergency department with a two-week history of sore throat, diffuse maculopapular rash, and migratory polyarthralgia. Two days prior to admission, she had been prescribed amoxicillin for these symptoms without improvement.

**Figure 1:**
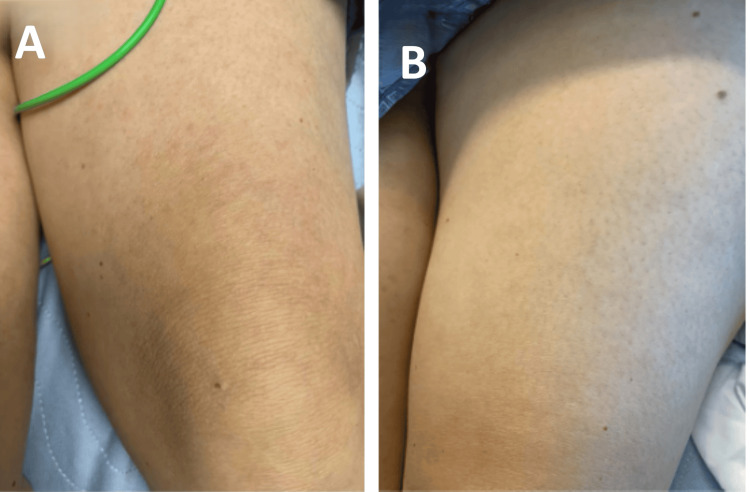
Faint, painless, maculopapular salmon-pink rash before use of steroids (A) and marked interval improvement after initiation of high-dose steroids (B)

On physical examination, she exhibited diffuse joint tenderness and a salmon-pink maculopapular rash distributed over the trunk and upper thighs (Figure 1A). Initial laboratory work-up revealed diabetic ketoacidosis (DKA), likely secondary to medication noncompliance. While DKA was managed accordingly in the intensive care unit, its contribution to the overall inflammatory picture was deemed minimal, as systemic symptoms persisted despite metabolic correction. She remained febrile with leukocytosis and was empirically started on broad-spectrum antibiotics. Despite treatment, the patient’s fever, rash, and sore throat persisted.

Imaging, including CT neck, ruled out peritonsillar abscess. Extensive infectious work-up was negative, including COVID-19 polymerase chain reaction (PCR), blood and urine cultures, mononucleosis screen, HIV, rapid strep test, antistreptolysin O (ASO) titer, and coccidioidomycosis serology. Autoimmune and hematologic investigations were also negative: antinuclear antibody (ANA), rheumatoid factor, and myeloma panel. Chest X-ray and abdominal/pelvic CT scans showed no infectious source, and hand X-rays did not support an erosive rheumatologic process. Inflammatory markers were significantly elevated: C-reactive protein (CRP) was 198 mg/L (reference range: 0-10 mg/L), and ferritin was 6,540 ng/mL (reference range: 12-300 ng/mL). Serum IgE was markedly elevated at 3,837 kU/L (normal ≤114 kU/L); IgA was mildly elevated, while IgG and IgM remained within normal limits.

**Figure 2:**
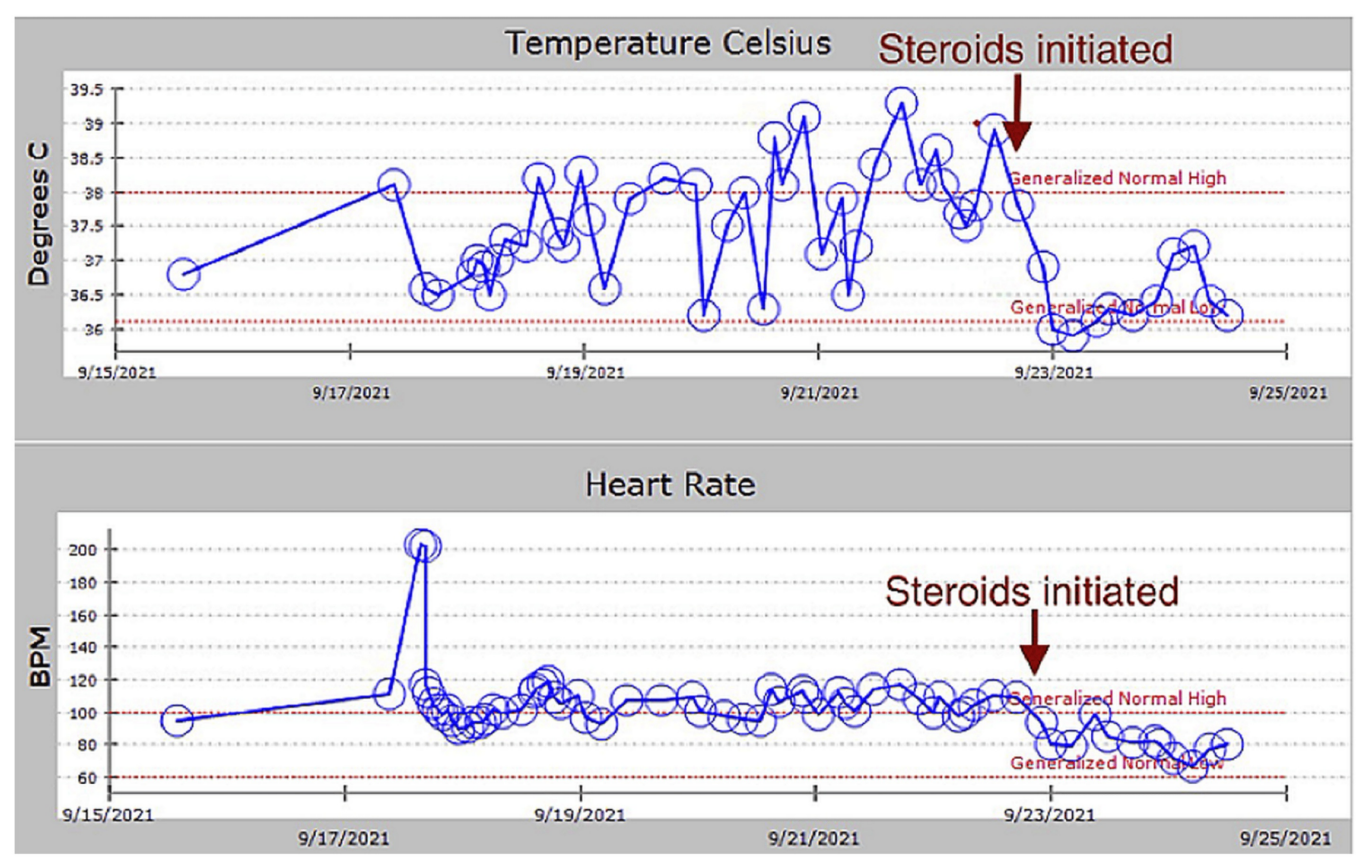
Immediate resolution of fever and tachycardia after initiation of high dose steroids

Given the absence of evidence for infectious, neoplastic, or autoimmune diseases such as systemic lupus erythematosus (SLE) or systemic vasculitis, and based on clinical findings and laboratory results, the patient met Yamaguchi’s criteria for AOSD. Broad-spectrum antibiotics were discontinued following Infectious Disease consultation. She was initiated on ibuprofen and high-dose prednisone, with a dramatic clinical response: resolution of fever, rash (Figure 1B), leukocytosis, tachycardia, arthralgia, and sore throat within 48 hours (Figure 2). The patient was discharged on high-dose prednisone with plans for outpatient Rheumatology follow-up to guide steroid taper and consideration of disease-modifying anti-rheumatic drugs (DMARDs) or biologics as steroid-sparing agents.

## Discussion

AOSD is a rare systemic inflammatory disorder consisting of fever, sore throat, arthralgia, a maculopapular rash, among other nonspecific symptoms, with elevated inflammatory markers. AOSD is more common in young adults. Some studies show that female individuals are more affected than males, but other studies show equal involvement [6]. The etiology of AOSD is unknown, but many studies have introduced various mechanisms, including genetic background, infectious triggers, activation of inflammation, and deficient resolution of inflammation [7,8]. The pathophysiology of AOSD is complex but involves the hyperactivation of macrophages and neutrophils, along with a dysregulated cytokine milieu [9,10].

Patients can develop three scenarios: (i) self-limited disease (single episode lasting two to 12 months), (ii) intermittent disease with recurrent flares and remissions, or (iii) chronic course with episodes lasting more than 12 months. Diagnosis is mostly clinical. The two widely used diagnostic criteria are Yamaguchi and Fautrel criteria [3,11-14]. Treatment options include monotherapy or combinations of non-steroidal anti-inflammatory drugs (NSAIDs), conventional DMARDs, and other biologics, depending on disease severity. Course and prognosis are not well defined, but some studies show that the chronic form has a worse prognosis than the self-limited and intermittent form [8]. In our case, the patient had fever, arthralgia, sore throat, and maculopapular rash, mimicking mononucleosis. She also had leukocytosis with neutrophilia, markedly elevated inflammatory markers, and a negative work-up for infectious etiology. The patient responded very well to steroid therapy.

As significant morbidity and mortality may result from AOSD, appropriate management of the disease in its infancy prior to its progression is the key to preventing complications like macrophage activation syndrome, a leading cause of mortality in patients with AOSD. Very high Ig-E levels, or hyperimmunoglobulin-E syndrome, are characterized by Ig-E levels > 1,500 IU/mL. It is usually described in genetic diseases, most commonly Job’s syndrome, characterized by immunodeficiency, recurrent skin and lung infections, and eczema [15,16]. Less commonly, such high levels of Ig-E can be associated with atopic and allergic diseases, helminthic infections, malignancies (e.g., Ig-E myeloma and some forms of Hodgkin’s lymphoma), and drug reactions [17,18]. In the current case, the patient exhibited markedly increased serum IgE concentrations during febrile episodes. She did not have any clinical or laboratory features to suggest a diagnosis known to be associated with hyperimmunoglobulin-E. She met criteria for AOSD and had a quick response to high-dose steroids as well. The occurrence of hyperimmunoglobulin-E in this case may represent an under-recognized immunologic response in a subset of AOSD patients. It is not listed in the diagnostic criteria, but the association might be under-recognized due to the rarity of this disease.

Increased concentrations of some immunoglobulins (especially IgG and IgM) are common during febrile episodes, but increases in IgE and IgA occur less frequently. Prior studies have described elevated IgG and IgM during febrile flares of AOSD, while IgA and IgE increases are less common. One hypothesis is that interleukin (IL)-4, a key regulator of IgE production, may contribute to hyper-IgE in AOSD, although the dominant cytokines implicated in AOSD pathogenesis remain IL-1, IL-6, IL-18, and TNF-α [9,10,19]. While the IL-4-related mechanism remains speculative, it warrants further investigation, especially in cases with marked eosinophilia or allergic features. Another study found that among 21 AOSD patients with persistent skin eruptions and eosinophilia, four patients were initially misdiagnosed as hyperimmunoglobulin-E syndrome due to overlapping features [20]. However, their IgE levels were not reported consistently, making direct comparisons to our case difficult. 

It is difficult to ascertain how common this association is, and whether hyperimmunoglobulin-E should be included in the diagnostic criteria of AOSD needs further studies. In the current case, IgE levels were not remeasured after treatment, which limits our ability to determine whether IgE trends correlate with disease activity. It might have some implications for treatment in the future, with the development of more targeted therapies for rheumatological diseases associated with acute febrile illness that mimic acute infectious processes. 

## Conclusions

AOSD is a rare but well-recognized cause of fever and nonspecific systemic symptoms, and remains a diagnosis of exclusion. Hyperimmunoglobulin-E, while not a classical feature of AOSD, may serve as a potential biomarker in atypical presentations. Further research is warranted. Although rare, this finding may assist in clinical suspicion and could prompt further exploration into the disease’s underlying immunologic mechanisms.
